# Clinical and Biochemical Characteristics of a Danish and Turkish Cohort of Incident and Prevalent Patients with Primary Biliary Cholangitis

**DOI:** 10.5152/tjg.2025.24300

**Published:** 2025-01-06

**Authors:** Hasan Eruzun, Lars Bossen, Dilara Turan Gökçe, İlkay Ergenç, Zekiye Nur Harput, Neslihan Güneş Aydemir, Meral Akdoğan Kayhan, Hasan Basri Yapıcı, Tugay Doğan, Peter Holland-Fischer, Jesper Bach Hansen, Yusuf Yılmaz, Orhan Sezgin, Haydar Adanır, Ahmet Bektaş, Henning Grønbæk

**Affiliations:** 1 Department of Gastroenterology, Ondokuz Mayıs University Faculty of Medicine, Samsun, Türkiye; 2 Department of Hepatology and Gastroenterology, Aarhus University Hospital and Clinical Institute, Aarhus, Denmark; 3 Department of Internal Medicine, Regional Hospital Horsens, Horsens, Denmark; 4 Department of Gastroenterology, Ankara Bilkent City Hospital, Ankara, Türkiye; 5 Department of Gastroenterology, Marmara University Faculty of Medicine, İstanbul, Türkiye; 6 King’s College Hospital Institute of Liver Studies, London, UK; 7 Department of Gastroenterology, Mersin University Faculty of Medicine, Mersin, Türkiye; 8 Department of Gastroenterology, Akdeniz University Faculty of Medicine, Antalya, Türkiye; 9 Marmara University Faculty of Medicine, İstanbul, Türkiye; 10 Department of Gastroenterology and Hepatology, Aalborg University Hospital, Aalborg, Denmark; 11 Department of Gastroenterology, Recep Tayyip Erdoğan University Faculty of Medicine, Rize, Türkiye

**Keywords:** Primary biliary cholangitis, Danish, Turkish, treatment response, ursodeoxycholic acid

## Abstract

**Background/Aims:**

Primary biliary cholangitis (PBC) is a chronic liver disease influenced by environmental, genetic, and epigenetic factors, with varying incidence across populations. This study compared demographic, biochemical, and treatment responses in Danish and Turkish PBC patients.

**Materials and Methods:**

Four cohorts were analyzed: (1) 101 incident Turkish patients, (2) 77 incident Danish patients, (3) 103 prevalent Turkish patients, and (4) 155 prevalent Danish patients. Demographics, biochemical data, disease severity, and response to ursodeoxycholic acid (UDCA) were assessed. Regression analysis was applied to the entire cohort to identify factors associated with the UDCA response.

**Results:**

Female dominance was noted in all cohorts (>90%) except the Danish incident group (75%). Turkish patients in the prevalent cohort were younger than Danish patients. Antimitochondrial antibody (AMA) positivity was higher in Turkish cohorts (92%-97%) compared to Danish cohorts (75%-88%). Biochemical data were similar across groups, with 15% of both populations having cirrhosis. Turkish patients showed a higher complete response to UDCA. Elevated alanine aminotransferase (ALT) levels reduced UDCA response, while Turkish ethnicity and older age improved it.

**Conclusion:**

Despite similar disease severity and age at diagnosis between Danish and Turkish PBC patients, Turkish patients had higher AMA positivity and better responses to UDCA treatment. These findings suggest potential genetic or environmental influences on treatment efficacy.

**Main Points**

- There are only a few studies regarding the course of disease and the response to UDCA in populations of primary biliary cholangitis from different genetic backgrounds.
- Our study comparing the ursodeoxycholic acid treatment responses and cirrhosis rates of Turkish and Danish PBC patients is valuable in this respect.
- While ursodeoxycholic acid response was higher in Turkish PBC patients, no difference was found in basic clinical and biochemical data, as well as cirrhosis rates.

## Introduction

Primary biliary cholangitis (PBC) is an autoimmune, inflammatory, and cholestatic liver disease with chronic non-suppurative cholangitis that primarily affects interlobular and septal bile ducts. Genetic risk factors and environmental triggers such as urinary tract infection and hormonal treatments may contribute to the ethiology.^1^ Ursodeoxycholic acid (UDCA) is the first-line treatment; however, up to 40% of patients are incomplete responders, leading to a higher risk of progression to cirrhosis or liver failure.^2,3^ Nevertheless, risk of liver transplantation due to PBC decreases with early diagnosis and treatment.^4,5^ Primary biliary cholangitis displays different prevalence and incidence in all races and regions; however, its prevalence is gradually increasing^2^ while data regarding the increase in incidence are debatable.^6-8^ The prevalence of PBC is higher in North America and Europe, while it is relatively low in the Asia Pacific region.^2^ Approximately 90% of patients with PBC are women, but a few register-based studies have shown a higher proportion of men affected by the disease. Moreover, the disease course in men is associated with a lower response rate to treatment, a higher risk of cirrhosis, and higher risk of liver-related complications than in women.^9^

Since genetic characteristics are important in the development of PBC, the course of the disease may differ in different races.^10,11^ In our study, we evaluated Turkish and Danish patients with incident and prevalent PBC in terms of age, gender, AMA positivity, and basic biochemical values. Further, we compared UDCA response rates and the cirrhosis frequency in patients with PBC from both populations. In addition, we evaluated the factors influencing UDCA response across the whole cohort.

## Material and Methods

### Study Populations

**Figure 1.**
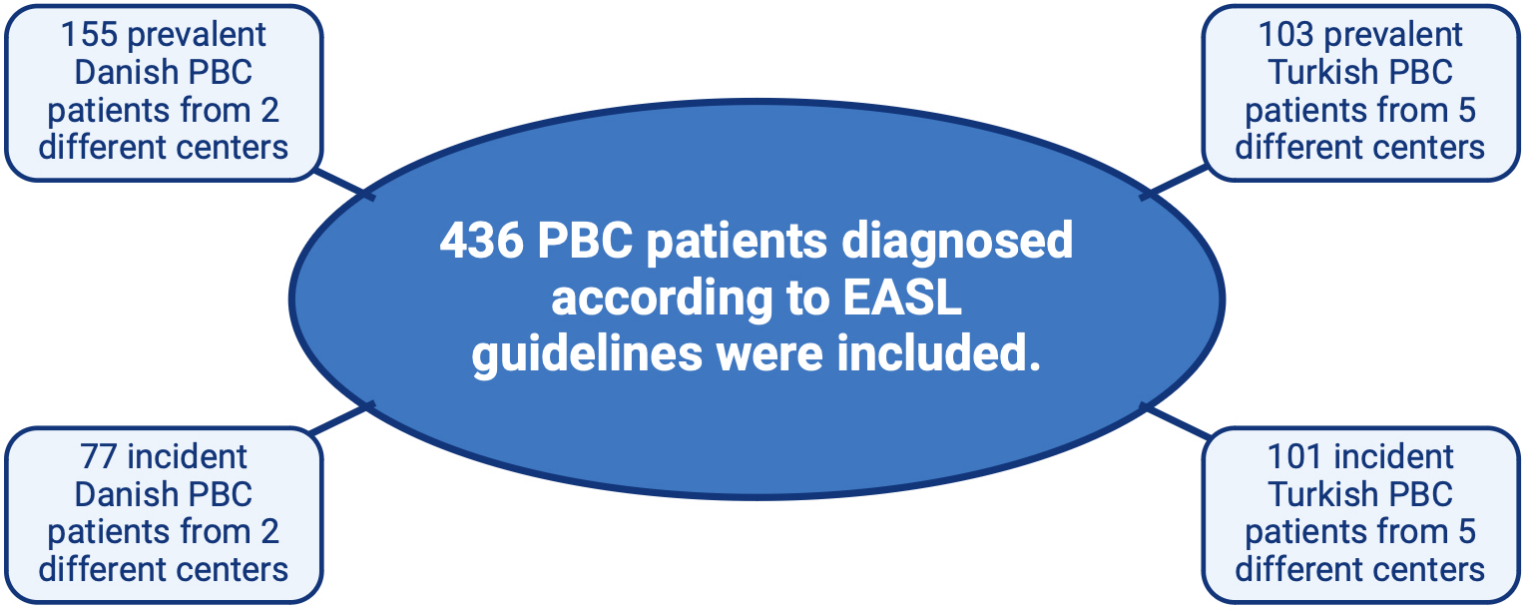
Flowchart of the study.

We retrospectively included 204 Turkish PBC patients, 103 prevalent and 101 incident patients, from 5 tertiary centers in Türkiye, followed between 2014 and 2022. Further, we recruited 232 Danish PBC patients, 155 prevalent and 77 incident patients, from 2 tertiary liver centers in Denmark (Figure 1). Thus, a total of 436 PBC patients were included in the study. Inclusion criteria were a diagnosis of PBC (prevalent or incident) with or without cirrhosis (with a few decompensated cirrhosis). Exclusion criteria were age under 18, life expectancy below 6 months, planned liver transplantation within 6 months, cirrhosis from other causes, liver cancer, and other malignancies for both groups. The inclusion period of prevalent Danish PBC patients was from September 2016 to April 2021, and from September 2016 to September 2021 for incident Danish patients with PBC. The Turkish patients who enrolled in the study were diagnosed between January 2014 and January 2021. The European Association for the Study of the Liver (EASL) 2017 guideline was used for the diagnosis of PBC.^12^ The Danish study was approved by the Ethics Committee of the Central Region of Denmark (approval number: 1-10-72-149-16, date: 22.09.2016). The Turkish study was approved by Ondokuz Mayıs University, Clinical Research Ethics Committee (approval number: 2023/223, date: 07.08.2023 ).

### Data Collection

For the Danish cohorts, data were collected as part of a clinical study protocol. Data about comorbidities, medications, previous decompensating events, as well as data from the time of diagnosis was collected from medical records (in the prevalent cohort). Turkish patients with PBC were recruited from hospital records in 5 different centers and a common cohort was created. Due to the retrospective nature of the study, it was determined that obtaining informed consent was not required. Patients were divided into groups as Danish prevalent/incident and Turkish prevalent/incident cases. In the first stage, gender, age, AMA positivity rate, ALP, ALT, platelet, total bilirubin, international normalised ratio (INR), and immunoglobuline M (IgM) levels were recorded. Cirrhosis rates, UDCA dose, and response rate were then determined. Ursodeoxycholic acid response was evaluated according to Toronto criteria after at least 12 months.^13,14^ where patients were categorized as incomplete responders if their ALP was above 1.67 times the upper limit of normal (ULN) of alkaline phosphatase (ALP) and/or their total bilirubin was above the upper limit of normal (ULN), and otherwise responders. Each country’s own laboratory limits were used for the ALP and total bilirubin upper limit. Patients were categorized as having cirrhosis if they had cirrhosis on liver biopsy, had liver stiffness above 16.9 kPa, had oesophageal varices, ascites, hepatic encephalopathy, or a cirrhotic liver on imaging.^15^

### Statistical Analysis

Data are reported as percentage or median with interquartile range (IQR). Independent sample *t*-test was used in the analysis of normally distributed variables, Mann–Whitney *U*-test was used in the analysis of non-normally distributed variables, and Pearson Chi-square test was used in intergroup comparisons of categorical variables. Univariate and multivariate regression analyses were conducted to examine the associations between specified variables and the outcome of interest. The cut-off *P*-value for statistical significance was taken as .05. Statistical analysis was performed in Statistical Package for Social Sciences (SPSS) version 20 (IBM SPSS Corp.; Armonk, NY, USA).

## Results

Clinical and biochemical data are shown in Tables 1 and 2 for the incident and prevalent cohorts, respectively. In Table 3, regression analyses of variables affecting treatment response for the entire cohort are presented. There was no difference in gender among prevalent patients, with 94% in the Danish and Turkish incident cohorts, respectively; however, the proportion of women was significantly higher in Turkish incident patients (93% vs. 75%, *P *= .02). Further, Turkish prevalent patients were younger than Danish prevalent patients, while there was no difference in age in incident patients.

The AMA positivity rate was high at the time of diagnosis in both incident (97%) and prevalent (92%) Turkish PBC patients compared with Danish incident (75%) and prevalent (88%) PBC patients (*P* : < .03). There was no significant difference in biochemical variables such as ALP, ALT, total bilirubin, INR, and IgM between Turkish and Danish incident and prevalent patients.

The UDCA treatment response rates at the 12th months are higher in Turkish prevalent patients in Table 2 (*P* = .006). A standard dose of 750 mg/day UDCA was used in Danish patients, while an individual doşe of 13-15 mg/kg was used in Turkish patients.

Cirrhosis rates in both Turkish and Danish patients were similar, around 15% for both incident and prevalent patients.

Univariate and multivariate analyses were conducted to examine the associations between various variables and the UDCA treatment response for all patients (Table 3). In the univariate analysis, age (OR = 1.035, 95% CI [1.011, 1.06], *P* = .004), ALT levels (OR = 0.957, 95% CI [0.943, 0.972], *P* < .001), and being Turkish were significantly associated with the outcome (OR = 2.25, 95% CI [1.247, 4.059], *P* = .007). However, in multivariate analysis, only ALT (OR = 0.961, 95% CI [0.945-0.977], *P* < .001) and being of Turkish origin (OR = 2.85, 95% CI [1.098-7.406], *P:* 0.031) were independently associated with UDCA response.

## Discussion

In this study, the diagnostic features, disease severity, treatments, and response to treatment of patients with PBC were evaluated in 2 populations from different regional and genetic backgrounds. Our main findings were that Turkish and Danish patients have similar age at diagnosis, and the same proportion have cirrhosis. However, Turkish patients have higher AMA positivity rates than Danish patients, and more Turkish patients than Danish patients have a complete response to UDCA treatment.

In our study, the proportion of women in the incident Danish cohort (75%) was lower than in the Turkish cohort (93%) patients but similar (both 94%) in the 2 prevalent cohorts. The female-to-male ratio in PBC patients varies by race, but there is no specific regional trend. Danish national registries show a ratio of 4.2:1 in Denmark, while this ratio is 6:1 in the United Kingdom and 2.3:1 in the Lombardia region of Italy, with a European average of 10:1 almost similar to the ratio of 9:1 in Japan.^16-18^ Previous studies show that the female-to-male ratio may vary between nations with similar genetic characteristics but might also exhibit similarities in nations with entirely different genetic backgrounds. In our study, the female-to-male ratios were comparable in the prevalent patient group in both countries; however, the male ratio among incident patients was higher in the Danish group. This finding aligns with the global trend of an increasing frequency of PBC in men.^19^ The prevalence of PBC worldwide tends to rise in men, influencing the female-to-male ratio, particularly in developed, industrialized Western societies and Japan.^19^ Nevertheless, recent Italian data contradict this trend, revealing a female-to-male ratio of around 2:1 in 2009, which increased to 4:1 in 2016.^19^ The higher male rate in Danish incident PBC patients may be attributed to the higher awareness among Danish physicians that the disease can also be seen in men and also that the Danish hospitals are tertiary referral centers seeing more challenging cases. In addition, in a Mediterranean society like Türkiye, the variability in the female-to-male ratio may have shown a similar trend as observed in Italy. However, there is a need for comprehensive studies on this subject to investigate the true prevalence and incidence of PBC in Turkish society and changes in incidence and prevalence over time.

In our study, the rate of AMA positivity in both prevalent and incident patients was found to be lower in the Danish population. This may be explained by an earlier policy to perform liver biopsy in AMA-negative Danish PBC patients to secure the diagnosis, while anti-Gp210 and anti-sp100 may be used more often in Türkiye.^20,21, 22^

Cirrhosis rates and the course of the disease appear to be similar between the 2 populations. There are few studies in the literature on the course of PBC and response to UDCA in different ethnicities. In a study by Levy et al^10^ in the United States, the risk of developing variceal bleeding, portal hypertension, and ascites was higher in PBC patients of Hispanic ethnicity than non-Hispanics. In the same study, being of Hispanic origin was associated with a poor response to UDCA treatment. In another study among PBC patients on the transplant waiting list, portal hypertension complications were higher in African Americans and Hispanics than in whites.^23^ Additionally, transplant rates for non-white patients were found to be lower and with longer time on the waiting list. It can be speculated that the worse course of the disease in African Americans and Hispanics might be due to limitations in access to health insurance and services as well as genetic background. In our study, cirrhosis rates were similar in the 2 countries with universal public insurance and similar and free access to health services.

In Denmark, when we initiated the study, the standard dose of UDCA in the treatment of PBC was 750 mg daily. This has since been changed and now Danish PBC patients are treated with the same dose as used in Türkiye with 13-15 mg/kg as the standard treatment dose. Unfortunately, the total UDCA dose was not recorded in Turkish patients, and we are unable to investigate the difference in total UDCA doses between the 2 countries. However, the UDCA response rate in Turkish patients was higher after 1 year using the Toronto criteria. Across the entire cohort, only lower ALT levels and Turkish ethnicity were independently associated with an increased likelihood of treatment response. In the above study by Levy C et al^10^, the UDCA response according to race was lower in people of Hispanic origin. However, another study conducted in the United States evaluated UDCA responses were evaluated in patients with PBC of White, Black, Latino, and other races and found no difference according to race.^24^ It is clear that more comprehensive studies are needed to evaluate UDCA response according to race. In our study, the response rate to treatment was lower in younger patients, consistent with the literature.^8^ The lack of a significant difference in the treatment response rate between men and women may be partly related to the small number of patients. Additionally, the association between elevated pre-treatment ALT levels and UDCA treatment non-response is consistent with the literature.^25^ In a study evaluating UDCA treatment response in PBC patients with autoimmune hepatitis features, ALT levels were higher in the non-responsive group.^26^ In our study, PBC patients with elevated baseline ALT levels may represent PBC variants with autoimmune hepatitis features. However, a more definitive interpretation could not be made due to the lack of data on anti-nuclear antibody (ANA) status and biopsy characteristics.

The major strength of the present study is the inclusion of a significant number of well-characterized incident and prevalent patients with PBC from 2 different ethnic populations. A weakness of the study is the retrospective design and the absence of total UDCA drug doses in the dataset, which may influence the interpretation of response rates. Furthermore, there could be potential bias introduced by differences in healthcare practices between the 2 countries.

In conclusion, our investigation of patients with PBC from 2 different genetic backgrounds showed that Turkish and Danish patients have similar age at diagnosis, and the same proportion have cirrhosis. However, Turkish patients have higher AMA positivity rates than Danish patients. Further, more Turkish patients than Danish patients have a complete response to UDCA treatment. More comprehensive studies examining patients with PBC from different races in detail regarding epidemiology, UDCA treatment response, and long-term follow-up are warranted.

## Lay Summary/Key Points

In this study, clinical and demographic data of PBC patients from 2 different genetic backgrounds were evaluated. Cirrhosis rates and treatment responses of PBC patients in Turkish and Danish populations were compared. Cirrhosis rates were similar in the 2 countries; however, the response rate to UDCA treatment was higher in Turkish PBC patients.

## Figures and Tables

**Table 1.** Demographic, Biochemical Data, and Treatment Response Rates of Danish and Turkish Incident PBC Patients

Variable	Danish Incident Patients n = 77	Turkish Incident Patients n = 101	*P*
Female, n (%) Female to male ratio	58 (75) 3 : 1	94 (93) 13 : 1	**.02**
Age years, median (IQR)	58 (51-66)	58 (46-62)	.75
AMA pos at diagnosis n (%)	67 (88)	98 (97)	**<.03**
ALP I/U, median (IQR)	236 (142-354)	286 (132-332)	.41
ALT U/L, median (IQR)	62 (38-103)	62 (31-92)	.78
Bilirubin µmol/L, median (IQR)	10 (6-14)	8 (7-11)	.16
INR, median (IQR)	1 (1-1.1)	1 (0.9-1)	.89
IgM g/L (0.4-2.3)	2.9 (1.7-4.3)	2.3 (1-3.1)	.32
Platelets 10^9^/L, median (IQR)	272 (218-325)	252 (192-303)	.54
Cirrhosis, n (%)	11 (14)	15 (14)	.08

pos, positivity; AMA, anti mitochondrial antibody; IQR, interquartile range.

**Table 2.** Demographic, Biochemical Data, and Treatment Response Rates of Danish and Turkish Prevalent PBC Patients

Variable	Danish Prevalent Patients n = 155	Turkish Prevalent Patients n = 103	*P*
Female, n (%) Female to male ratio	146 (94) 16:1	97 (94) 16 : 1	.9
Age years, median (IQR)	61 (51-70)	54 (51-67)	**.0001**
AMA-pos at diagnosis n (%)	110 (75)	95 (92)	**<.03**
ALP I/U, median (IQR)	146 (108-217)	176 (101-190)	.51
ALT U/L, median (IQR)	32 (22-49)	37 (19-46)	.6
Platelets 10^9^/L, median (IQR)	257 (189-309)	253 (191-311)	.2
Bilirubin µmol/L, median (IQR)	8 (6-12)	13 (7-14)	.17
INR, median (IQR)	1 (1-1)	0.9 (0.9-1)	.21
IgM g/L (0.4-2.3)	2.4 (1.4-3.7)	3.8 (1.6-5.4)	.18
Cirrhosis, n (%)	24 (16)	16 (16)	.41
UDCA dose	750 mg (750-750)	13-15 mg/kg	–
UDCA responders at 12 months, n (%)	84 (65)	88 (85)	**.006**

pos, positivity; AMA, anti mitochondrial antibody; IQR, interquartile range; UDCA, ursodeoxycholic acid.

**Table 3.** Regression Analysis of Response to UDCA Treatment for Whole Cohort

	Univariate	*P*	Multivariate	*P*
	OR [95% CI]		OR [95% CI]	
Age	1.035 [1.011-1.06]	**.004**		
Male gender	0.737 [0.238-2.277]	.596		
Cirrhosis	0.768 [0.385-1.534]	.455		
Total bilirubin	0.768 [0.53-1.11]	.162		
ALT	0.957 [0.943-0.972]	**< .001**	0.961 [0.945-0.977]	**< .001**
AMA	0.907 [0.45-1.827]	.785		
Turkish Nation	2.25 [1.247-4.059]	**.007**	2.85 [1.098-7.406]	**.031**
IgM	0.951 [0.803-1.125]	.555		

AMA, anti mitochondrial antibody; OR, odds ratio.

## Data Availability

The data that support the findings of this study are available on request from the corresponding author. Since it was a retrospective study, informed consent was not obtained from the patients.
